# 

**Published:** 1893-10

**Authors:** 


### CONTENTS.

ORIGINAL COMMUNICATIONS—

- A Few Clinical Notes on the Usual Situation of Spinal Hemorrhage, which Results from Traumatic Influence, with Report of Four Typical Cases. By Thomas H. Manley, M.D., New York .......... 449
- Enterectomy for Obstructive Epithelioma at the Ileo-Cecal Valve; Secondary Anastomosis Operation by Abbé's Long Incision. By James M. Barton, A.M., M.D., Philadelphia .......... 456
- Syphilitic Condylomata of the Auditory Canal. By Charles Dunbar Roy, A.B., M.D., Atlanta, Ga. .......... 461
- An Approximate Estimate of the Energy Contained in Strychnine. By A. D. Barr, M.D., Calamine, Ark .......... 466

SOCIETY REPORTS—

- Transactions Atlanta Obstetrical Society .......... 469

EDITORIAL—

- To Subscribers .......... 478
- The Fever at Brunswick .......... 479
- Pan-American Medical Congress .......... 480
- Medical Library for the State .......... 481

SELECTIONS AND ABSTRACTS—

- Skin Diseases and Syphilis .......... 483
- Obstetrics and Gynecology .......... 487
- Surgery .......... 494
- General Medicine and Therapeutics .......... 499
- Eye, Ear, Nose and Throat .......... 506

MEDICAL ITEMS .......... 509

ADVERTISERS' INDEX TO ADVERTISEMENTS .......... xii

CLASSIFIED INDEX TO ADVERTISEMENTS .......... xlii

ITEMS OF INTEREST .......... x, xi, xii

PREMIUM OFFERS .......... xv

